# Tuning proton-coupled electron transfer by crystal orientation for efficient water oxidization on double perovskite oxides

**DOI:** 10.1038/s41467-020-17657-9

**Published:** 2020-08-27

**Authors:** Yunmin Zhu, Zuyun He, YongMan Choi, Huijun Chen, Xiaobao Li, Bote Zhao, Yi Yu, Hui Zhang, Kelsey A. Stoerzinger, Zhenxing Feng, Yan Chen, Meilin Liu

**Affiliations:** 1 State Key Laboratory of Pulp and Paper Engineering, School of Environment and Energy, South China University of Technology, Guangzhou, 510000 China; 2 College of Photonics, National Chiao Tung University, Tainan, 71150 Taiwan; 3 State Key Laboratory of Functional Materials for Informatics, Shanghai Institute of Microsystem and Information Technology, Chinese Academy of Sciences, Shanghai, 200050 China; 4 Materials Science and Engineering, Georgia Institute of Technology, Atlanta, GA 30314 USA; 5 School of Physical Science and Technology, ShanghaiTech University, Shanghai, 201210 China; 6 School of Chemical, Biological and Environmental Engineering, Oregon State University, Corvallis, OR 97331 USA

**Keywords:** Catalytic mechanisms, Energy, Electrocatalysis

## Abstract

Developing highly efficient and cost-effective oxygen evolution reaction (OER) electrocatalysts is critical for many energy devices. While regulating the proton-coupled electron transfer (PCET) process via introducing additive into the system has been reported effective in promoting OER activity, controlling the PCET process by tuning the intrinsic material properties remains a challenging task. In this work, we take double perovskite oxide PrBa_0.5_Sr_0.5_Co_1.5_Fe_0.5_O_5+δ_ (PBSCF) as a model system to demonstrate enhancing OER activity through the promotion of PCET by tuning the crystal orientation and correlated proton diffusion. OER kinetics on PBSCF thin films with (100), (110), and (111) orientation, deposited on single crystal LaAlO_3_ substrates, were investigated using electrochemical measurements, density functional theory (DFT) calculations, and synchrotron-based near ambient X-ray photoelectron spectroscopy. The results clearly show that the OER activity and the ease of deprotonation depend on orientation and follow the order of (100) > (110) > (111). Correlated with OER activity, proton diffusion is found to be the fastest in the (100) film, followed by (110) and (111) films. Our results point out a way of boosting PCET and OER activity, which can also be successfully applied to a wide range of crucial applications in green energy and environment.

## Abstract (web summary)

Proton-coupled electron transfer (PCET) has been observed in chemical, energy, and biological transformation processes. Here we demonstrate that the rate of PCET and oxygen evolution reaction can be dramatically enhanced by tuning crystal orientation and the correlated proton diffusion.

## Introduction

Proton-coupled electron transfer (PCET)^1^ processes have been observed in a wide variety of key reactions in energy^2^, environmental^3^ and biological^4^ systems. Among them, the oxygen evolution reaction (OER) has received intensive investigation since the development of cost-effective and high-performance catalysts for OER is crucial for the practical applications of various devices for chemical and energy transformation, such as fuel cells and water electrolyzers^5–7^. The most commonly used noble metal-based OER catalysts (i.e., RuO_2_ and IrO_2_) may not be appropriate for the large-scale applications owing to their high cost and poor stability. Transition metal oxides have potential to replace noble metal catalysts due to their low cost and high stability^1,8–10^. However, in order to meet the increasingly demanding requirements for practical applications, the catalytic activity of transition metal oxides needs to be further improved.

A simplistic picture of the OER involves four subsequent PCET processes. In reality, however, the proton transfer and electron transfer may occur sequentially, resulting in decoupled transfer of proton and electron in the reaction^11^. It has been demonstrated that the OER activity of metal oxides can be promoted strongly by speeding up the proton transfer and hence promoting the PCET process. For example, Yamaguchi et al.^12^ tuned the interfacial proton transfer by adding pyridine and its derivatives into the electrolytes, resulting in a significant improvement in the OER activity. A similar approach was adapted by Takashima et al.^13^ to accelerate the PCET on hematite, which reduced the overpotential of OER by about 250 mV. Yang et al.^14^ modified the surface of the La_1−x_Sr_x_CoO_3−δ_ with phosphate ion groups (Pi), which was considered to enhance the interfacial proton transfer, thereby increasing the OER activity of the perovskite material. While these examples demonstrated the potential improvement of the OER activity of metal oxides by the acceleration of the PCET process, introducing additives into the solution or to the material surface may have drawbacks as well, such as increasing the system complexity and compromising the long-term stability. It is critical to gain deeper understanding of the key factors that determine the intrinsic properties of the catalysts to dramatically accelerate the PCET and improve the OER activity.

Several recent works demonstrated that the rate of ionic diffusion (oxygen ion or proton) in the bulk phase can critically impact the OER kinetics at the surfaces^15–18^. For example, She et al.^17^ reported that by introducing proton acceptor Sr_3_B_2_O_6_ into various perovskite-based oxides, including LaCoO_3−δ_, La_0.4_Sr_0.6_CoO_3−δ_, Pr_0.5_Ba_0.5_CoO_3−δ_, Ba_0.5_Sr_0.5_Co_0.8_FeO_3−δ_, and Sr_0.8_Co_0.8_Fe_0.2_O_3−δ_, OER activities can be significantly improved, which is attributed to the facilitated deprotonation process. Similar effects in promoting proton transfer and corresponding OER activity were observed by Takashima et al.^18^ when they introduced solid proton acceptor La_2_O_3_ into α-Fe_2_O_3_. These pioneering studies suggest that it is possible to tune the PCET process and improve the OER activity by regulating the proton transport properties in materials. To prove the strategy, perovskite-oxides with intrinsic proton conductivity offers the ideal model system^19–24^. Furthermore, the rate of ionic diffusion in perovskite oxides depends strongly on the crystal orientation, and the difference can be up to orders of magnitude^25–27^. Therefore, examining the dependence of OER kinetics on the crystal orientation of a proton conductor can provide critical information about the effect of proton conductivity on PCET and OER activity.

In this work, the double perovskite oxide PrBa_0.5_Sr_0.5_Co_1.5_Fe_0.5_O_5+δ_ (PBSCF) is chosen as a model system because of its outstanding OER activity^28,29^, attributed to the excellent conductivity of proton, oxygen ion, and electrons or ‘triple’ conductivity (H^+^/O^2−^/e^−^)^19–22^. PBSCF thin films with well controlled surface orientation of (100), (110), and (111) are prepared on appropriate single crystal substrates (LaAlO_3_) using pulsed laser deposition (PLD). Electrochemical measurements and DFT based calculations consistently verify that the OER activity of PBSCF thin films follows the order of (100) > (110) > (111). The high OER activity of the (100) surface is attributed to its facilitated deprotonation process, leading to an effective PCET process as observed in our experiments and confirmed by theoretical analysis. In addition, synchrotron-based near ambient pressure X-ray photoelectron spectroscopy (APXPS) clearly reveals that water molecules are deprotonated to surface hydroxyl species the most effective on the (100) surface. Correlated with the high OER activity and facilitated proton transfer process, the proton diffusivity is corroborated with the same order of (100) > (110) > (111). All these results evidently demonstrate that the PCET process and OER activity of double-perovskite electrocatalysts can be practically controlled by the crystal orientations, affecting subsequent proton diffusion. This approach can be widely applied for guiding the rational design of high-performance electrocatalysts in significant green energy and environmental applications.

## Results

### Synthesis of PBSCF thin films with different orientations

**Fig. 1:**
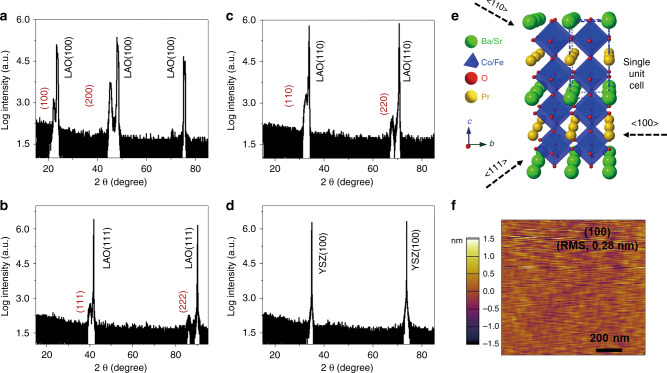
Thin film characterization. **a**–**d** HRXRD 2θ–ω scans of a PBSCF (100) film on LAO (100) (**a**) a PBSCF (111) film on LAO (111) (**b**) a PBSCF (110) film on LAO (110) (**c**) and a polycrystalline PBSCF thin film on YSZ (100) (**d**). **e** A schematic of the crystal structure of PBSCF. The arrows indicate the directions of <100>, <110>, and <111>; **f** AFM images of a PBSCF (100) thin film.

By choosing appropriate single crystal substrates, epitaxial or highly textured oxide thin films with specific surface orientation can be efficiently grown by PLD techniques^25,29–31^. For PBSCF, the X-ray diffraction (XRD) peaks of (110) and (102) planes overlap (Supplementary Fig. 1), indicating that the lattice parameter c is very close to 2a_p_^19,28^. In this work, LaAlO_3_ (100), LaAlO_3_ (110), and LaAlO_3_ (111) single crystals were used as the substrates for PLD deposition of PBSCF thin films. High resolution X-ray diffraction (HRXRD) results showed that the PBSCF thin films grown by PLD were highly textured. For simplicity, hereafter LaAlO_3_ refers to as LAO. As shown in Fig. 1a–c, PBSCF thin films grown on LAO (100), LAO (110), and LAO (111) have surface orientations of (100), (110) and (111), respectively^32^. For comparison, HRXRD results of polycrystalline PBSCF thin films (Supplementary Fig. 1a) grown on a inert Yttrium stabilized ZrO2 (YSZ) (100) single crystal substrate are depicted in Fig. 1d, which shows only substrate peaks in the 2θ–ω scans^28,29^. Fig. 1e displays the crystal structure of PBSCF with a double perovskite structure^28,33^. The arrows in Fig. 1e denote the direction of <100>, <110>, and <111>.

The surface morphology of obtained PBSCF thin films was measured by atomic force microscopy (AFM). Figure 1f shows a representative AFM image for the (100) film. All of the PBSCF thin films exhibit a very smooth surface with a surface roughness of ~ 0.5 nanometer (Supplementary Fig. 2). The thin film thickness of PBSCF is ~ 100 nm (Supplementary Fig. 3). The surface composition of the obtained PBSCF thin films was probed by X-ray photoelectron spectroscopy (XPS). No noticeable difference was observed in surface composition for the films with different orientations. (Supplementary Figs. 4 and 5).

HRXRD, AFM, and XPS results suggest that we have obtained highly textured PBSCF (100), (110) and (111) thin films (hereafter referred to as (100), (110), and (111), respectively) with ultra-smooth surfaces and similar surface composition. The use of the model systems could avoid the potential effect of microstructure and chemical composition so that we can focus on understanding of the influence of crystal orientation on the electrochemical performance.

### Impact of orientation on OER activities of PBSCF thin films

OER activities of the obtained PBSCF thin films with different orientations were systematically evaluated in a standard three-electrode cell configuration with the thin films as the working electrode, Ag/AgCl as the reference electrode, and Pt as the counter electrode. A thin gold layer was sputtered on the PBSCF thin films to serve as the current collector. In order to avoid the influence of the current collector on the OER, the inactive area was waterproofed before experiments (Supplementary Fig. 6). As shown in Supplementary Fig. 7, the LAO substrate and Au current collector exhibit negligible OER activities.

**Fig. 2:**
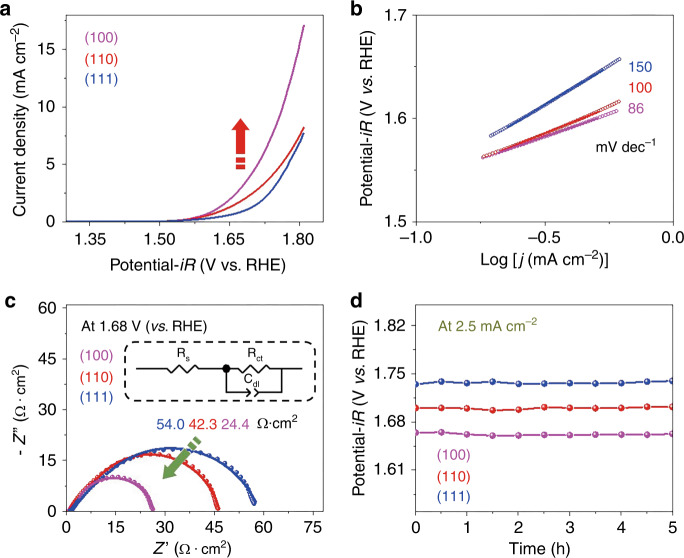
OER activity for PBSCF thin films with different orientation. **a** LSV curves; **b** Tafel plots; **c** Nyquist plots of OER on PBSCF thin-film electrodes in 1 M KOH at 1.68 V (vs. RHE; solid dots are the raw data, solid lines are the fitted results, and the inset figure shows the electrical equivalent circuit); and **d** Chronopotentiometry curves at a constant current density of 2.5 mA cm^−2^.

The linear sweep voltammetry (LSV) of PBSCF thin films with different orientation was performed in 1 M KOH (Fig. 2a). The observed overpotential for the (100) film at a current density of 2.5 mA cm^−2^ was 434 mV, smaller than those for the (110) and (111) films (468 mV and 504 mV, respectively). In addition, at 1.7 V (vs. RHE), the (100) film exhibited a current density of 4.3 mA cm^−2^, higher than those for the (110) and (111) films (2.5 mA cm^−2^ and 1.3 mA cm^−2^, respectively). These results clearly verify that the OER activity of the PBSCF thin films depends on its surface orientation, and the (100) film shows the highest OER activity. Consistently, the Tafel slopes for the (100) film (86 mV dec^−1^) are much lower than those for the (110) and (111) films (100 and 150 mV dec^−1^, respectively) (Fig. 2b). It is noted that the Tafel slopes of PBSCF thin films obtained in this study are larger than those for polycrystalline PBSCF nanofibers prepared by electrospinning^28^. The difference in Tafel slopes is attributed to the variation in surface crystal orientations, surface microstructures, and defect states of the samples used. Furthermore, the electrochemical impedance spectroscopy (EIS) spectra of all the films show a semicircle in the Nyquist plot (Fig. 2c), which can be fitted by an OER charge transfer resistance (*R*_ct_), and a total ohmic resistance (*R*_s_) of the test system and a constant phase element^34,35^. While the *R*_s_ for all of the films is quite similar, the *R*_ct_ shows a strong dependence on the surface orientation. The *R*_ct_ of the (100) film is the smallest, suggesting that the (100) thin film has the fastest OER reaction kinetics^36^. The EIS spectra collected at different potentials (Supplementary Fig. 8a–c) also show that the *R*_ct_ value of the (111) film is the highest, followed by the (110) and (100) films for all the potential values. For instance, at 1.77 V (vs. RHE), the *R*_ct_ value of the (111) film is 3.1 times as high as that of (100). All these electrochemical measurements clearly demonstrate that the OER activity of the PBSCF films depends sensitively on the surface orientation (i.e., (100) > (110) > (111)).

In addition to the electrocatalytic activity measurements, we also carried out long-term stability tests. Shown in Fig. 2d are the chronopotentiometry curves of the PBSCF films with different orientations at a constant current density of 2.5 mA cm^−2^. All of the films demonstrated excellent stability without performance degradation. The (100) film consistently exhibits the lowest overpotential. Moreover, scanning electron microscope (SEM) images verify that no significant change in the surface morphology of the PBSCF thin films was observed even after the long-term electrochemical tests (Supplementary Fig. 9). Furthermore, HRXRD results show that after the electrochemical tests, all of the films are still highly textured with the same surface orientation as prepared (Supplementary Fig. 10a–c). Similar to the activity examination, even after the long-term stability tests, the (100) film still exhibits the highest OER activity.

**Fig. 3:**
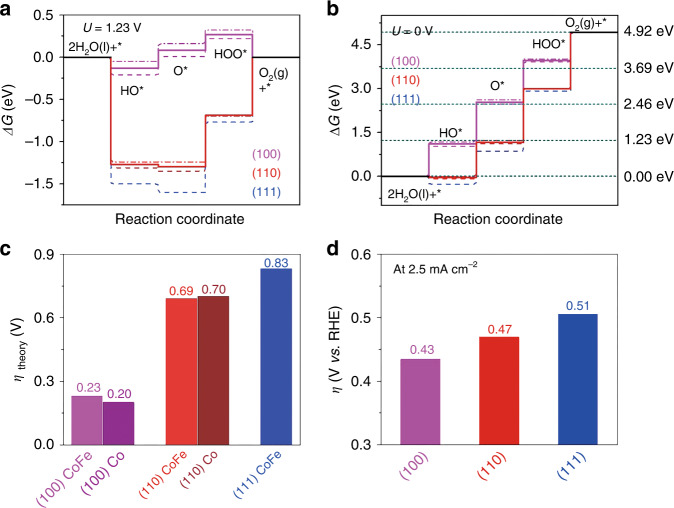
DFT calculations investigating OER activities for PBSCF. **a**, **b** Gibbs free energy diagram for OER on PBSCF (100), (110), and (111) surfaces **a** at U = 1.23 V **b** and at U = 0.0 V. Dashed lines are Co-terminated surfaces, while dash-dotted are CoFe-terminated. Solid lines for (100) and (110) surfaces represent its averaged Gibbs free energies. Short dashed lines in dark cyan color are the Gibbs free energies for OER with the ideal concerted proton and electron process; **c** Calculated overpotentials for PBSCF with different orientation and surface termination; **d** Overpotentials extracted from LSV curves at a current density of 2.5 mA cm^−2^.

To gain more insight into the effect of PBSCF’s surface orientation on OER activity, we performed DFT calculations. Since B site cations are normally considered to be the active site for the OER in perovskite-based oxides_,_ we constructed a simplified model system of PBSCF surfaces with a BO−terminated layer (Supplementary Fig. 11)^8,15,16,28,37^. For the PBSCF thin films with different orientations, we observed little difference in surface composition, suggesting that the OER activity is relatively insensitive to surface termination (Supplementary Fig. 5), as confirmed by DFT-based mechanistic studies. Representative adsorption configurations for O*, HO*, and HOO* on (100) is shown in Supplementary Fig. 11g. The Gibbs free energy diagrams of the four-step OER for PBSCF with different surface orientations are shown in Fig. 3a and b for *U* = 1.23 and *U* = 0, respectively. Compared to the (110) and (111) surfaces, (100) appears to show smaller overpotential, suggesting the highest OER activity. In addition, the potential determining step (PDS) for the (100) is found to be HO* → O*, while the PDS for the (110) and (111) surfaces is identified to be HOO* → * + O_2_ and O* → HOO*, respectively (Fig. 3a). The Gibbs free energy change in the PDS for the (100) is 0.23 eV, which is much smaller than 0.69 eV and 0.83 eV for the (110) and (111) surfaces, respectively (Supplementary Table 1). As summarized in Supplementary Table 2, the adsorption energies of oxygen (E_ad,O*_) are well correlated with ΔG_O*_ – ΔG_HO*._ The variation of theoretical overpotentials for the OER demonstrates that the (110) and (111) surfaces have larger overpotentials than the (100) (Fig. 3c), which is qualitatively in agreement with experiments (Fig. 3d). The overpotential at the OER current density of 2.5 mA cm^−2^ for (100) is lower than that of (110) and (111) (i.e., 0.43 V versus 0.47 V and 0.51 V, respectively).

### Deprotonation process and proton diffusion on PBSCF surfaces

While the electrochemical test and the DFT calculation in the previous section clearly demonstrated that the surface orientation critically impact the OER activities, it is still unclear whether such differences were arisen from the different proton transfer process in OER. It has been widely reported that the dependence of the OER reaction kinetics on the proton activity can be derived from the following equation:

1 $$\rho ^{{\mathrm{RHE}}} = \left( {\frac{{\partial {\mathrm{log}}(i)}}{{\partial {\mathrm{pH}}}}} \right)_{E^{{\mathrm{RHE}}}}$$

where ρ^RHE^ is the proton reaction order^11,14,17^. When the deprotonation processes are strongly coupled with an electron transfer, its OER kinetics shows little dependence on the pH value of a solution (vs. RHE), leading to a low ρ^RHE^ value. It was reported that LaCoO_3_ follows the concerted proton-electron transfer process^14,15,38^. In contrast, if the deprotonation process is involved in the PDS along with decoupling of the electron transfer process, the OER kinetics depends strongly on pH with a larger ρ^RHE^ value. For instance, it was observed that various mixed ionic-electronic conductors (MIECs) (i.e., =La_0.5_Sr_0.5_CoO_3_ and Pr_0.5_Ba_0.5_CoO_3_) have lattice oxygen participation in the OER, leading to a decoupled proton-electron transfer process in OER and a strong pH dependence^15,17,38,39^.

**Fig. 4:**
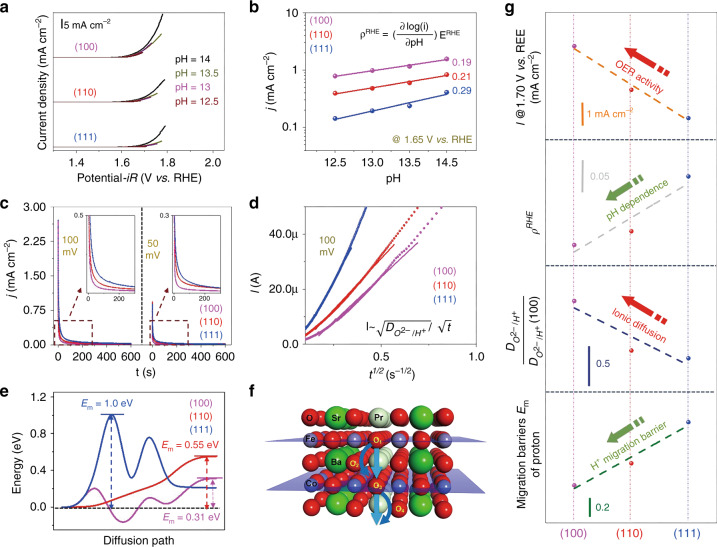
OER kinetics at different pH and ionic diffusion characteristics. **a** LSV curves for the PBSCF thin films measured in O_2_-saturated KOH with pH = 12.5 to 14. **b** OER current at 1.65 V versus RHE plotted in log scale as a function of pH for the (100), (110), and (111) thin films. **c** Chronoamperograms of the (100), (110) and (111) thin films at a constant bias of 100 mV and 50 mV vs. Hg/HgO. **d** Measured current *I* as a function of *t*^−*1/2*^. **e** Energy profiles for proton diffusion in bulk PBSCF with different crystallographic directions. **f** Geometrical illustrations of oxygen and proton bulk diffusion in PBSCF along (100). **g** Comparison of the OER activity, pH dependence, ionic diffusivity, and migration barriers E_m_ of protons of (100), (110), and (111).

To gain a deeper understanding of the OER kinetics on PBSCF thin films, we investigated the OER activities for PBSCF thin films at different pH values in detail. LSV curves for the (100), (110), and (111) films at the pH values of 12.5, 13, 13.5 and 14 are shown in Fig. 4a. Figure 4b displays the OER currents in log scale as a function of pH, from which the extracted ρ^RHE^ value for the (100), (110) and (111) films are 0.19, 0.21 and 0.29, respectively. For comparison, Supplementary Fig. 12a shows the ρ^RHE^ values for various perovskite-based oxides, including LaCoO_3_, La_0.5_Sr_0.5_CoO_3−δ_^14^, PrBaCoO_3_^15^. All of the PBSCF thin films exhibit a noticeably higher ρ^RHE^ value than that for LaCoO_3_, suggesting a non-negligible contribution to the proton transfer process in the potential determining step. Interestingly, by changing of the surface orientation from (111) to (110) and (100), the ρ^RHE^ value (i.e., pH dependence) decreases, which may indicate the occurrence of a facilitated proton transfer process on the (100) surface. As reported, OER activities can be strongly enhanced by improving the coupling of electron and proton transfers on the electrocatalyst surfaces, as depicted in Supplementary Fig. 12b^14,17^. Therefore, it is highly plausible that the (100) film surface may exhibit interfacial proton transfer properties, leading to a concerted proton-electron transfer process. Accordingly, such difference develops a better coupled electron-proton transfer process on the (100) film in OER than the (110) and (111) films, which consequently results in the different OER activities in the order of (100) > (110) > (111) as shown in Fig. 2.

As demonstrated above, PBSCF thin films with different surface orientations exhibit quantitatively different behavior in proton-coupled electron transfer processes. Therefore, its careful rationalization may be imperative to fully understand the mechanism. As mentioned in the introduction section, a number of recent works reported that by introducing a proton acceptor in the bulk materials, the deprotonation process can be strongly facilitated, resulting in improved OER activities^17,18^. To further understand the mechanism for the difference in OER activity of PBSCF thin films, the ionic diffusion characteristics in the PBSCF thin films were experimentally taken into account.

The ionic diffusion in PBSCF thin films with different orientation was characterized chronoamperometrically based on a bounded 3-D diffusion model^40,41^. A small cathodic potential step was applied to the PBSCF thin films, and then the current was measured as a function of relaxation time. As shown in Fig. 4c, the current decreases noticeably faster on the (100) film, suggesting its higher ionic diffusivity than (110) and (111) films^40,41^. All of the PBSCF films were prepared and measured under the same experimental conditions. The films were sufficiently thick to rule out the impact of elastic strain introduced by the substrate^42,43^. Therefore, the relative ion diffusion coefficients of the films with different orientation can be extracted from the slope of the linear region in *I* vs. *t*^*−1/2*^ curves^40,41^ as shown in Fig. 4d. The (100) film shows the highest ionic diffusivity, followed by (110) and (111) (Fig. 4g). In general, protons may transport in both through-plane and in-plane directions. In this study, however, the ionic diffusion probed by the chronoamperometric measurements is dominated by the diffusion in the through-plane direction, because the film in the in-plane direction can be considered under the same electrochemical potential for protons at a given electric bias. Due to its triple conducting-oxide characteristics, the carrier for the ionic diffusion in PBSCF can be ideally both oxygen ions and protons^19,21,22^. As reported, a strongly enhanced OER activity can be achieved by doping LaCoO_3_ with strontium (Sr) ions, which was attributed to its higher oxygen ion diffusivity than undoped LaCoO_3_^15,16^. A stronger pH dependence, however, was observed in Sr-doped LaCoO_3_ than LaCoO_3_, which is attributed to a lattice oxygen mechanism. In contrast, we observed that the (100) film has the highest ionic diffusivity and OER activity, but with the lowest pH dependence among three thin film samples studied. In addition, according to our simulated results shown in below, the migration barriers for oxygen ions are significantly higher than those for protons (Supplementary Table 3). As a result, the proton diffusion is expected to occur much more easily than the oxygen ionic diffusion. Therefore, we believe that the higher OER activity of the (100) film is due likely to its higher proton conductivity along the <100> directions, which may facilitate the proton transfer process. To further reveal the impact of crystal orientation on the ionic diffusion, we carried out DFT calcuations to evaluated the diffusion barriers of proton and oxygen ions in bulk PBSCF (Supplementary Fig. 13) and to examine their diffusion characteristics as discussed above (Fig. 4e). Supplementary Table 3 shows that the migration barriers of oxygen ions are much higher than those of protons, demonstrating the effect of oxygen ions is negligible under the experimental conditions at 25 ^o^C. Figure 4e displays the energy profiles of diffusion of protons with different crystallographic directions. The energy increase of the (110) surface indicates a smooth proton transfer from a saddle point to its adjacent site without a barrier, while (100) and (111) surfaces have energy barriers before reaching a final saddle point. Figure 4f, g demonstrate the proton diffusion in (100) occurs much faster than that in (110) and (111). Figure 4e, g show the calculated energy barriers for protron diffusion along different crystal orientations, suggesting that diffusion along the [100] direction occurs more easily than [110] and [111]. Similar to the link between bulk oxygen diffusion and the ability to form surface oxygen vacancies^15–18^, we hypothesize that bulk proton transport similarly reflects the ability to remove protons from a given site on the surface. This computational finding supports the higher OER activity of (100) thin film than (110) and (111) may be orientated from the facilitated proton transfer via [100] directions.

Overall, the combination of our computational and experimental results confirm that the (100) has a better coupling between electron and proton processes than the (110) and (111), resulting in higher OER activities and lower overpotentials for OER.

### Deprotonation process on PBSCF surface studied by APXPS

**Fig. 5:**
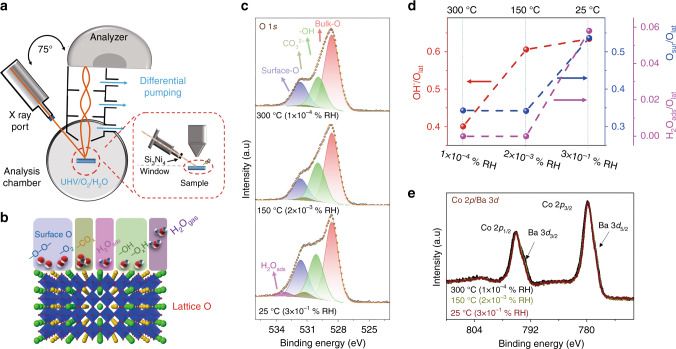
Adsorption of water on PBSCF (100) measured by APXPS. **a** Schematic of the APXPS. **b** Schematic illustration of the multilayer O model. **c** O 1s XPS spectra. **d** Comparison of fitting results for HO^−^/O_lat_, O_sur_/O_lat_ and H_2_O_ads_/O_lat_ ratio. **e** Co 2p/Ba 3d XPS spectra on the (100) film in 1 mbar H_2_O at 300 °C (1 × 10^−4^ % RH), 150 °C (2 × 10^−3^ % RH), and 25 °C (3 × 10^−1^ % RH).

The OER kinetics is determined primarily by the interaction between the electrocatalyst surface and the water molecules in the vicinity of the surface. To probe the adsorption properties of the PBSCF surfaces, we carried out XPS experiments under conditions of near ambient pressure and elevated temperature using a specially designed APXPS. Figure 5a schematically shows the experimental arrangement for the APXPS analysis in this study^44^, in which a gas doser, a laser heater and a thermal couple are not shown for simplicity. Before the measurements, PBSCF thin films were annealed in 1 mbar O_2_ gas environment at 300 °C for 30 min to remove most adsorbates, such as hydrocarbon or water molecules on the surface. During the XPS measurement, the amount of water molecules adsorbed on the film surfaces was controlled by changing the relative humidity (RH) in the chamber. While keeping the water vapor pressure (pH_2_O) in the chamber at 1 mbar, the sample temperature was varied from 300 °C, 150 °C, and 25 °C, resulting in RH value near the sample surface from 0.0001%, 0.002%, and 0.3%, respectively. The XPS data shown in the following section were collected using an X-ray with photon energy of 1150 eV.

Firstly, we probed the evolution of oxygen species on PBSCF surfaces as a function of RH. As water adsorption occurs on the transition metal oxides, various surface oxygen species were reported, including water molecules and surface hydroxyl and oxygen species (*-O, -O-O-*, etc.) (Fig. 5b)^45^. Shown in Fig. 5c are the O 1s XPS spectra on the (100) surface at different RH. All the O 1s spectra were fitted by five peaks, including bulk oxygen (Bulk-O) at ~ 528.6 eV, the hydroxyl group (-OH) at ~ 530.1 eV, hydrocarbon (-CO_x_) at ~ 531.2 eV, surface oxygen (Surface-O) at ~ 531.6 eV, and water molecules (H_2_O_ads_) at ~ 533.3 eV^46–48^. The -CO_x_ peaks were negligible at 300 °C, and showed small contribution at lower temperature due to the adsorption of residue carbon in the UHV chamber (Supplementary Fig. 14a). As the RH increases (i.e., temperature decreased), the intensity of -OH, Surface-O, and H_2_O_ads_ peaks become more pronounced (Fig. 5c). This trend can be observed more clearly from Fig. 5d, where the intensity ratios of the -OH peak, Surface-O, and H_2_O_ads_ peaks to the Bulk-O peak are plotted as a function of RH. We also noticed a similar behavior on (110) and (111) films (Supplementary Fig. 15).

Water molecules can be adsorbed on transition metal oxides via physical adsorption, dissociative adsorption, and a wide range of redox reactions^45,49^. In addition, if oxygen vacancies are present on the surfaces, water molecules can combine with oxygen vacancies, forming a hydroxyl group on the surface (i.e., H_2_O + V_o_^..^ → 2OH^−^)^49^. Water molecules could also go through oxidative adsorption processes; for example, water molecules deprotonate to form -OH, which is then further oxidized to different types of surface oxygen species (*-O, *-O^−^O-*, etc.)^45,50–52^. In this work, the RHs were controlled by changing the sample temperature from 300 ^o^C to 25 ^o^C while maintaining the water vapor pressure at 1 mbar. We did not observe any changes in Co 2p (Fig. 5e) and Fe 2p XPS spectra (Supplementary Fig. 16a) under different RH conditions, suggesting no formation of extra oxygen vacancies. Therefore, the increase of hydroxyl group with RH was not due to the presence of oxygen vacancies on the surface. Considering that PBSCF is a good water oxidation catalyst^28,29^, it is more likely that the interaction of water molecules with PBSCF surfaces went through oxidative adsorption processes^45,50–52^. Under higher RH conditions, more water molecules can be adsorbed onto PBSCF surfaces, as evidenced by the higher H_2_O_ads_ groups at higher RH. The water molecules can go through deprotonation process to form terminal hydroxyl groups of M-OH (M is normally metal sites), and then different types of surface peroxide species. As a result, we observed both the increase of -OH peaks and Surface-O peaks as shown in Fig. 5c, d.

The surface cation compositions were also quantified under different RH conditions, which showed negligible changes. The surface-related components in Sr increase with RH (Supplementary Fig. 14b)^25,53,54^ due to the binding to surface hydroxyl groups from water oxidative dissociation and residual -CO_3_ groups on the surface. Other cations showed negligible change with RH values, as shown in the Pr 3d, Co 3p/Fe 3p XPS peaks in Supplementary Fig. 16c, d. Similar results were observed for the (110) and (111) thin films (Supplementary Figs. 17 and 18).

**Fig. 6:**
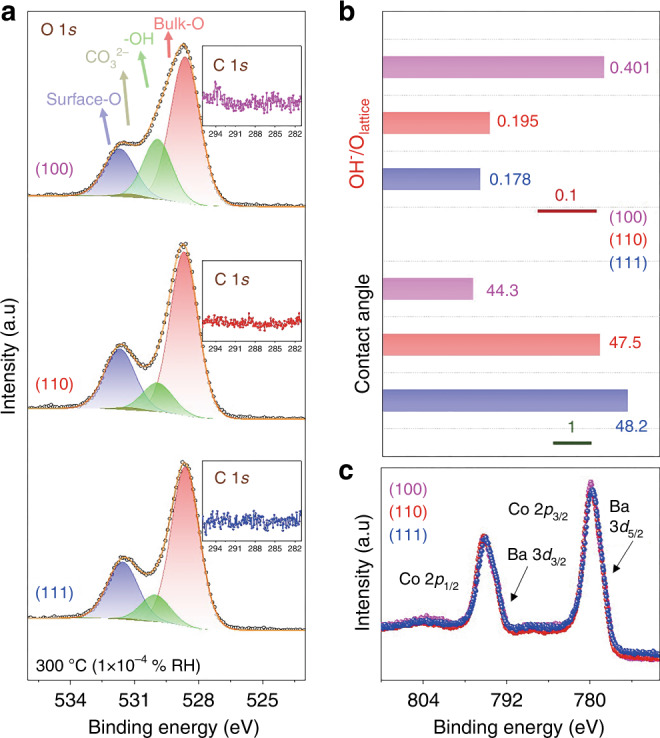
Surface adsorbate coverage on different surface orientation. **a** O 1s, (**c**) Co 2p/Ba 3d spectra for (100), (110) and (111) thin films in 1 mbar H_2_O at 300 °C (1 × 10^−4^ % RH). **b** Comparison of HO^−^/O_lat_ ratio for (100), (110) and (111) thin film in 1 mbar H_2_O at 300 °C (1 × 10^−4^ % RH), Contact angle for (100), (110), and (111) thin films (the scales bar was marked inside the figure).

Having shown that APXPS can provide information about the amount of active oxygen species, we further compared the relative amounts of these surface oxygen species on the PBSCF surface with different orientations at the same RH condition. To avoid complexity from surface carbon, the spectra in Fig. 6 were collected at 300 °C in 1 mbar water vapor pressure, in which the carbon contamination on all of the film surfaces is negligible (inset figure in Fig. 6a). It can be seen from O 1s XPS spectra (Fig. 6a) that the OH- peak (green peak) on the (100) film was noticeably higher than on (110) and (111) films. Its difference can be observed more clearly from Fig. 6b, where the -OH and Bulk-O peak ratio is plotted as a function of surface orientation. The OH-/O_lattice_ ratio followed the order of (100) > (110) > (111). The results under other RH conditions also follow the same trend with OH amount; (100) > (110) > (111), as shown in Supplementary Fig. 19. In addition, no significant change in the Co 2p/Ba 3d and Fe 2p XPS spectra were observed before and after the OER experiments (Supplementary Fig. 20). These results clearly suggest that while the fast proton path in the (100) film can facilitate the interface proton transfer during OER, these protons did not introduce noticeable changes in the films’ bulk properties. In addition, it was found that surface Sr content on the (100) surface is the highest among the three samples studied; this is consistent with its most surface hydroxyl groups as observed in the O 1s spectra^53,54^. The XPS spectra of other cations, such as Co 2p/Ba 3d (Fig. 6c), Fe 2p (Supplementary Fig. 21a), Pr 3d (Supplementary Fig. 21c), Ba 4d (Supplementary Fig. 21d) and Co 3p/Fe 3p (Supplementary Fig. 21e) were very similar to the surfaces with different orientation, which were in agreement with the ex situ XPS results shown in the previous section (Supplementary Fig. 5).

The amount of the hydroxyl group was reported to be closely related to the wettability of the surface^47,55^. We further tested the contact angle of electrolyte (1 M KOH) on the PBSCF thin films with different orientations. Figure 6b and Supplementary Fig. 22 show the contact angle relevant data of the electrolyte solution on differently oriented thin films. The contact angles on the (100) film was found to be the smallest (Fig. 6b and Supplementary Fig. 22), and the contact angle of the (110) film is between that of the (100) and (111) films. This result is in good agreement with the APXPS data that the amount of the hydroxyl group on the surface follow the order of (100) > (110) > (111) at a given RH condition. These results suggest that under a given RH condition (i.e., same temperature and P_H2O_), the PBSCF surfaces with different orientations exhibit a similar chemical composition with very different water absorption characteristics. Water molecules tended to become dissociated into the OH group more readily on the (100) surface.

In summary, our systematic studies clearly manifested that tuning of the proton transfer process can be effectively made through controlling of crystal orientations and associated ionic diffusion processes. Since proton transfer processes are widely involved in many important chemical and energy transformation processes^56–58^, our approach is applicable to these reactions. For example, we also examined the impact of crystal orientation on the hydrogen evolution reaction (HER) activity of three PBSCF thin films. As shown in Supplementary Figs. 8d–f and 23, it was found that the HER performance trend is the same as the OER (i.e., (100) > (110) > (111)). Similar to the OER, we believe that one significant contribution to the higher HER activity on the PBSCF (100) thin film than (110) and (111) is also due to the different proton transfer process on the surface as supported by using the adsorption energies of hydrogen (Supplementary Table 2).

## Discussion

In this work, we demonstrated that the proton-coupled electron transfer can be promoted by tuning of the crystal orientation and correlated proton diffusion, leading to strongly boosted OER activities. PBSCF thin films with (100), (110) and (111) surface orientation were synthesized by PLD on LAO substrates with appropriate orientation. Electrochemical measurements clearly showed that the OER activity of PBSCF thin films strongly depends on the surface orientation and follows the order of (100) > (110) > (111). In particular, such difference in OER activity can be sustained even after a long-term durability test. DFT calculations-based studies also supported the experimental findings. The highest OER activity for the (100) film was believed to be related to its coupled proton and electron transfer process as observed both experimentally and theoretically. Consistently, synchrotron-based APXPS results demonstrated that water molecules become deprotonated into surface hydroxyl species most effectively on PBSCF (100). Furthermore, correlated with the high OER activity and facilitated proton transfer process, the ionic diffusivity was found to follow the same order of (100) > (110) > (111). Our systematic studies pointed out boosting the OER activity of electrocatalysts can be successfully achieved by accelerating of the proton-coupled electron transfer process via controlling the crystal orientation and related ionic diffusion. It is strongly expected that our efficient tuning approach for catalytic activities can be effectively applied to a wide range of other energy and environment applications, including solar fuel productions and fuel cells^59^.

## Methods

### Pulsed laser deposition

Highly textured PBSCF thin films were grown onto single crystal LAO (100), LAO (110), LAO (111), and YSZ (100) substrates at 500 °C under an oxygen pressure of 10 mTorr with a KrF excimer laser of 248 nm wavelength at a pulse repetition frequency of 5 Hz and 10 Hz. After deposition, the thin film was cooled to room temperature at a cooling rate of 5 °C min^−1^ under an oxygen pressure of 2 Torr. The detail information about the PLD target synthesis can be found in the Supplementary Note 1.

### Preparation of thin film electrode

A gold current collector was deposited on the surface of the thin film using a sputter prior to the preparation of the test electrode. Subsequently, an Ag wire (Sigma, 99.99%) was fixed with Ag paint and cured in a 140 °C furnace for one hour. The current collector of the electrodes, the back, sides and the wires of electrodes are covered with a non-conductive, chemically resistant epoxy resin (E-44AB) that cures at room temperature for 24 h. This waterproofing treatment was carried out three times to ensure only the sample surface with fixed area (2.5 × 2.5 mm) were exposed to the electrolytes (Supplementary Fig. 6).

### Electrochemical measurements

Relevant electrochemical tests were performed using CH Instruments (CHI 660E, China). All electrolytes were prepared using deionized water (>18 MΩ cm^−1^) and KOH. A scan rate of 10 mV s^−1^ was used for all linear-scan voltammetry (OER and HER). Before to electrochemical tests, the electrolyte was O_2_-saturated for more than 30 min. To ensure O_2_/H_2_O equilibrium at 1.23 V vs. RHE, high purity oxygen was bubbled into the electrolyte during the measurement to saturate O_2_ (with details in the Supplementary Note 2).

### Characterization of the catalysts

The surface morphology of the PBSCF thin films was observed by SEM (SU-8010, Japan) and AFM (MFP-3D-S, USA). The surface chemistry was characterized by XPS using Thermo Fisher K-alpha (USA). The APXPS experiments were carried out at Beamline 02B01 (XPS endstation) of the Shanghai Synchrotron Radiation Facility (SSRF). The APXPS experiments were performed with the approval of the Proposal Assessing Committee of SiP.ME2 platform project (with details in the Supplementary Note 3). For ex-situ XPS, we used C 1s (284.6 eV) for calibration binding energies. For APXPS, because the C 1 response at 300 ^o^C is very weak, we use O 1s (Bulk-O, 528.6 eV) for calibration. The wettability of the surface of the thin film electrode was characterized by contact angle measurement with a 1 M KOH electrolyte (DSA-25, Germany). The crystal structure of the powder and polycrystal thin film was examined by XRD using a Bruker D8 Advance (Germany) with monochromated Cu K_α1_ radiation. The crystal structure of the highly textured thin film samples was characterized using HRXRD (DH1, United Kingdom).

### Density functional theory calculations

We applied the Vienna ab initio simulation package (VASP)^60^ with the projector-augmented-wave (PAW) method^61,62^. We also used the spin-polarization method with the Perdew-Burke-Ernzerhof (PBE)^63^ exchange-correlation functional. To accurately support the experimental findings, we first prepared a bulk model for PrBa_0.5_Sr_0.5_Co_1.5_Fe_0.5_O_6_ (PBSCF; Pr_4_Ba_2_Sr_2_Co_6_Fe_2_O_24_; *P4/mmm*) (Supplementary Fig. 11). The detail information about DFT calculations can be found in the Supplementary Note 4.

## Supplementary information

Supplementary Information

## Data Availability

The data that support the findings of this study are available from the corresponding author on request. Source data are provided with this paper.

## Source data

Source Data
