# Supplementary material for: Tuning proton-coupled electron transfer by crystal orientation for efficient water oxidization on double perovskite oxides
Source: Nat Commun. 2020 Aug 27;11:4299. doi: 10.1038/s41467-020-17657-9 (PMC7453016; doi:10.1038/s41467-020-17657-9)
Supplement: Supplementary file 1 — Supplementary Information [file 41467_2020_17657_MOESM1_ESM.pdf]

Supplementary Information

Tuning proton-coupled electron transfer by crystal orientation for efficient water oxidization on double perovskite oxides

Yunmin Zhu[1], Zuyun He[1], YongMan Choi[2*], Huijun Chen[1], Xiaobao Li[3], Bote Zhao[4], Yi Yu[3], Hui Zhang[3], Kelsey A. Stoerzinger[5], Zhenxing Feng[5], Yan Chen[1*] and Meilin Liu[4*]

[1]State Key Laboratory of Pulp and Paper Engineering, School of Environment and Energy, South China University of Technology, Guangzhou 510000, P. R. China. [2]College of Photonics, National Chiao Tung University, Tainan 71150, Taiwan. [3]State Key Laboratory of Functional Materials for Informatics, Shanghai Institute of Microsystem and Information Technology, Chinese Academy of Sciences, Shanghai 200050, China. [4]Materials Science and Engineering, Georgia Institute of Technology, Atlanta, GA 30314, USA. [5] School of Physical Science and Technology, ShanghaiTech University, Shanghai 201210, China. [6]School of Chemical, Biological and Environmental Engineering, Oregon State University, Corvallis, OR 97331, USA
e-mail: escheny@scut.edu.cn, ymchoi@nctu.edu.tw, meilin.liu@mse.gatech.edu

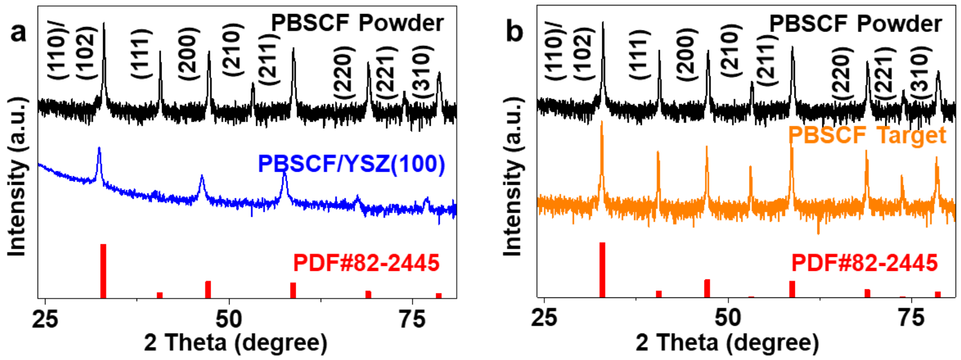

Supplementary Figure 1 XRD patterns of PBSCF **samples a** PBSCF powder and PBSCF/YSZ (100); **b** PBSCF powder and PBSCF target.

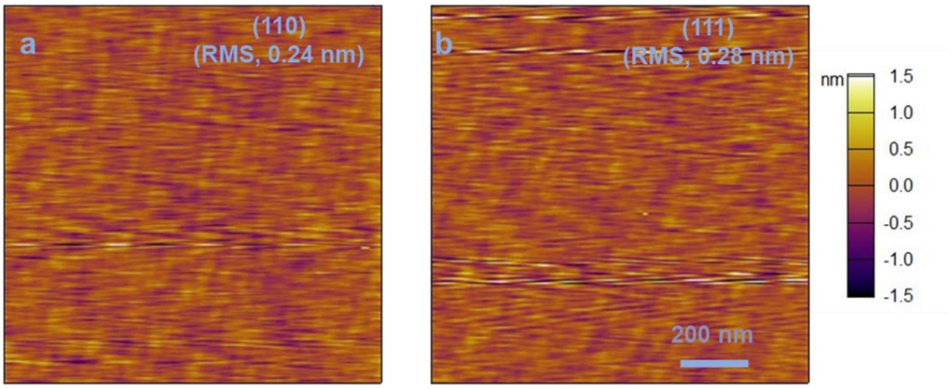

Supplementary Figure 2 AFM images of PBSCF thin film a (110) and **b** (111) thin film.

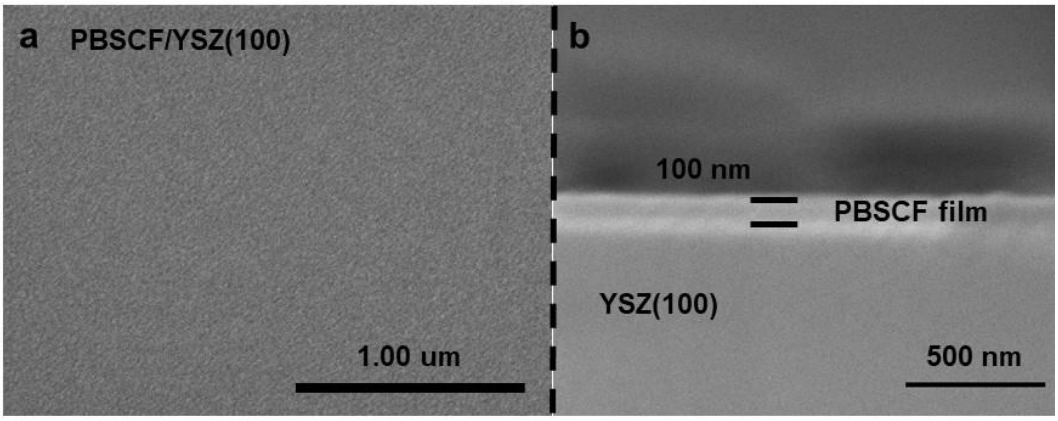

Supplementary Figure 3 SEM micrographs of PBSCF/YSZ (100) thin film
a surface; **b** cross-sectional

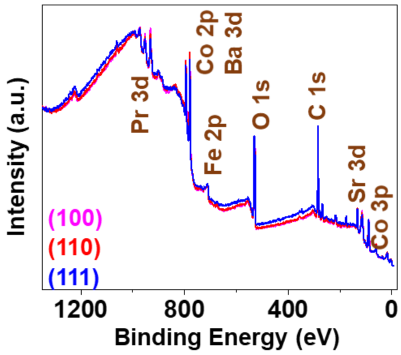

Supplementary Figure 4 XPS survey spectra of PBSCF thin films

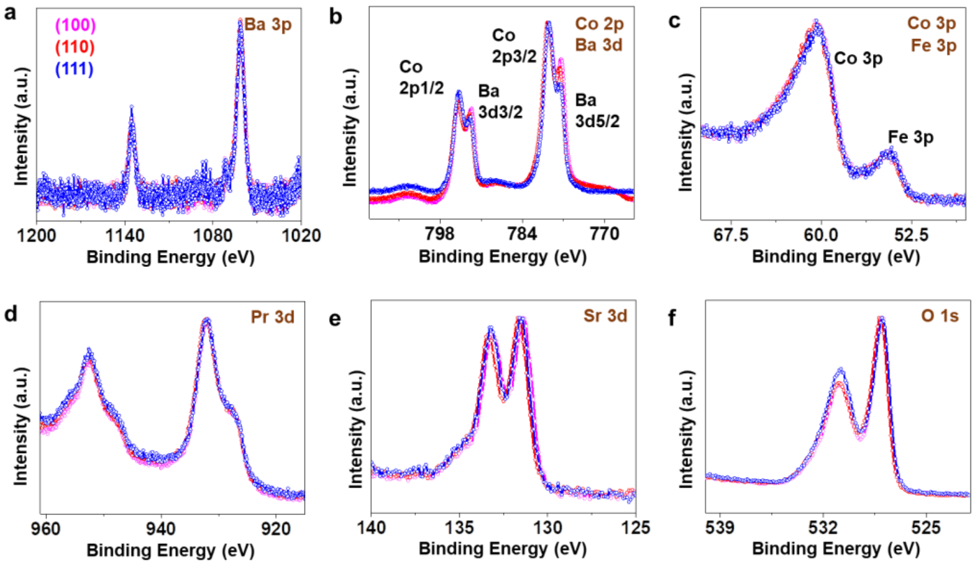

Supplementary Figure 5 XPS spectra of PBSCF thin films with different orientation a Ba 3p; **b** Co 2p/Ba 3d; **c** Co 3p/Fe 3p; **d** Pr 3d; **e** Sr 3d; and **f** O 1s.

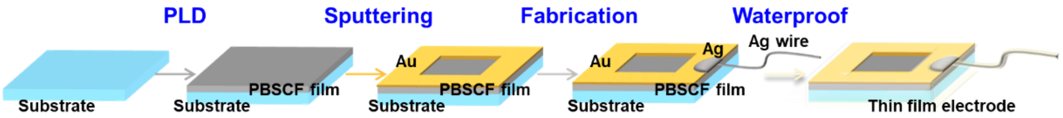

Supplementary Figure 6 Illustration of the sample preparation process for PBSCF thin film electrodes.

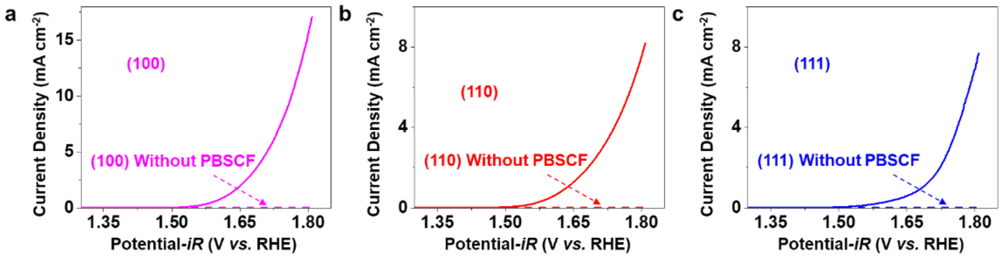

Supplementary Figure 7 LSV curves of $LaAlO_3$ substrates with and without PBSCF thin film a (100); **b** (110) **c** (110).

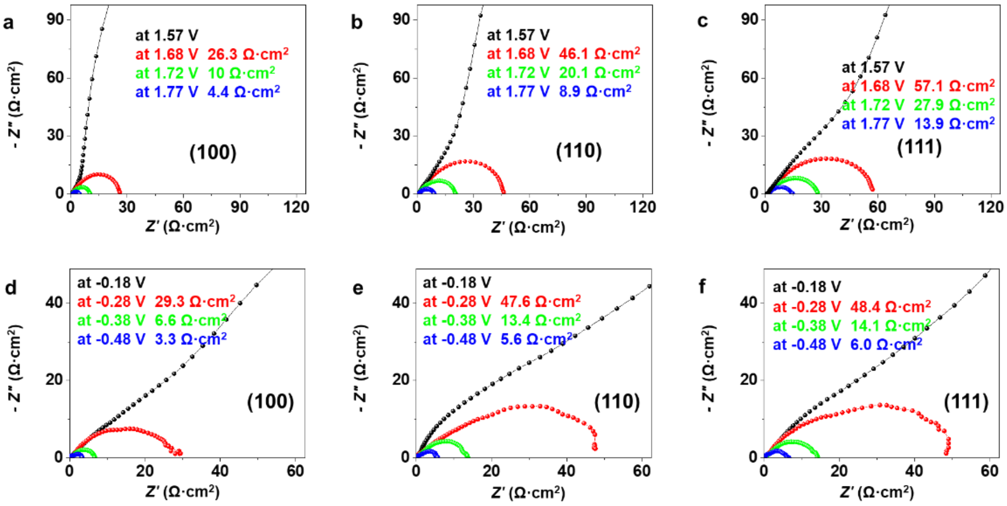

Supplementary Figure 8 OER and HER activity for PBSCF thin films with different orientation a-c and **d-f** Nyquist plots of OER and HER on PBSCF thin film electrodes in 1M KOH at 1.57 V, 1.68 V, 1.72 V, and 1.77 V; at -0.18 V, -0.28 V, -0.38 V, and -0.48 V (vs. RHE).

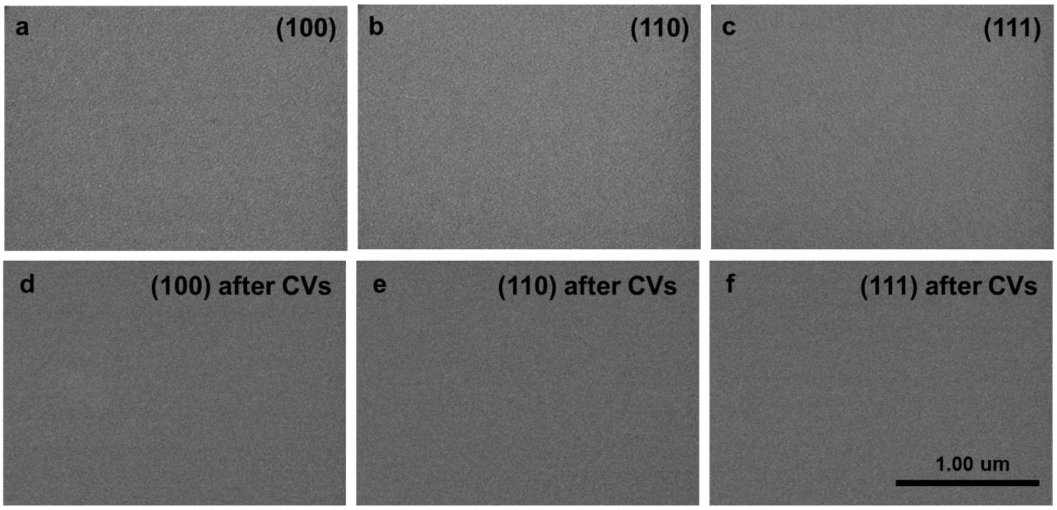

Supplementary Figure 9 SEM images of PBSCF thin films before and after CVs test a-c before CVs; **d-f** after CVs.

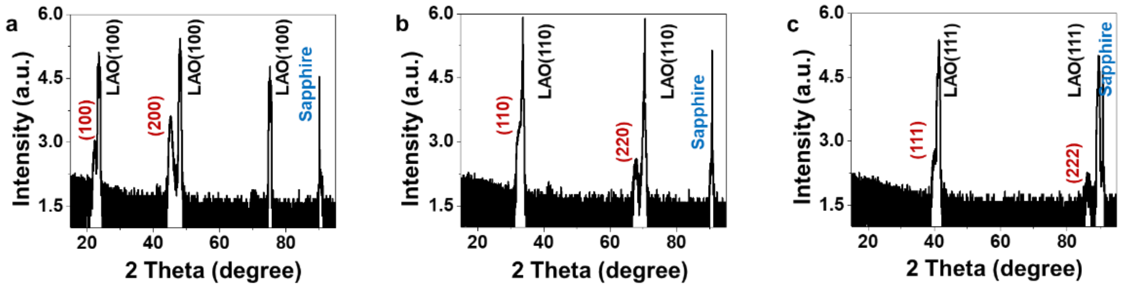

Supplementary Figure 10 HRXRD 2θ–ω scans of PBSCF thin films after CVs test
a PBSCF/LAO (100), **b** PBSCF/LAO (110) and **c** PBSCF/LAO (111)

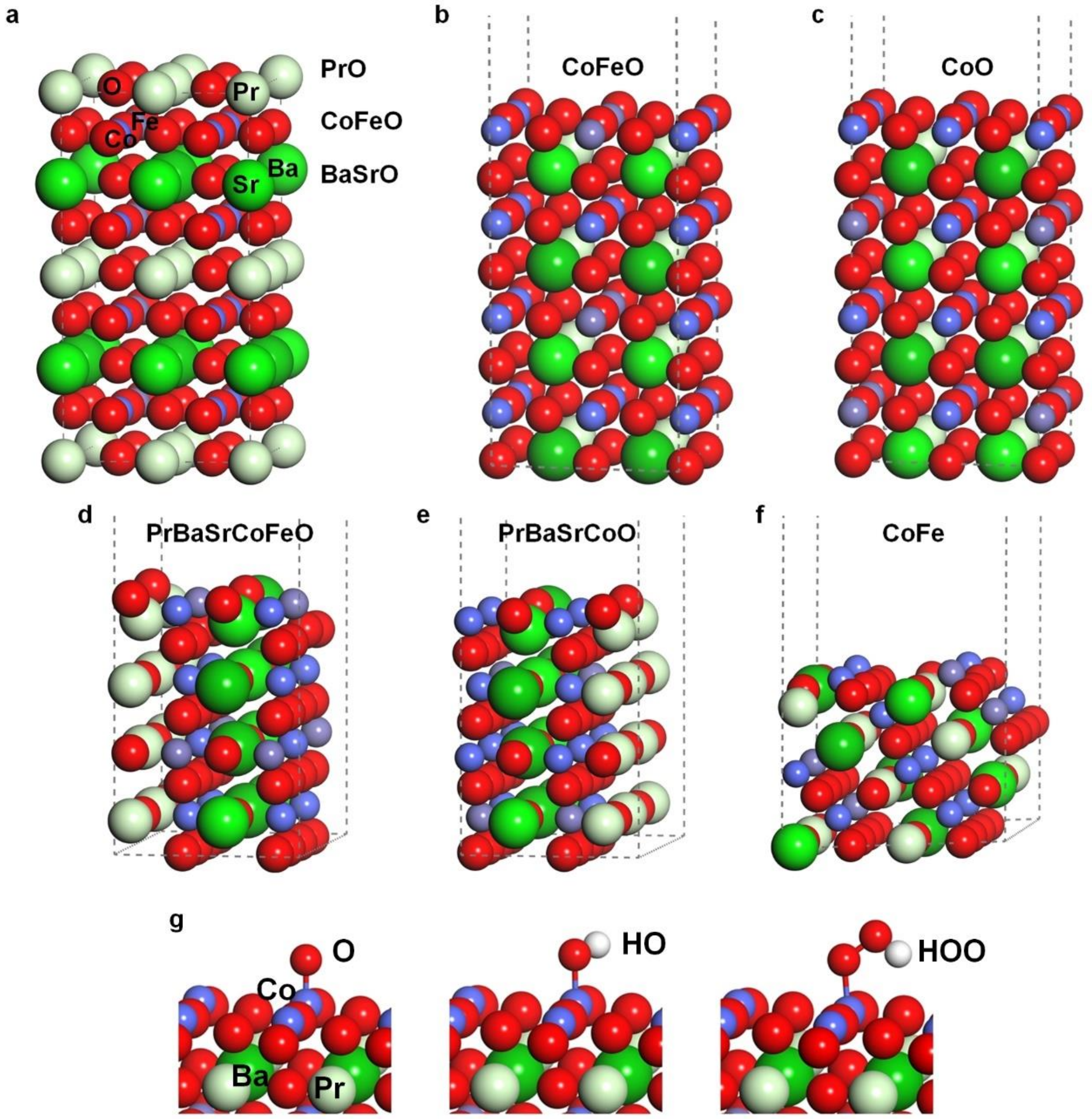

Supplementary Figure 11 Bulk structure and surface model for DFT calculations
a The bulk structure of a PBSCF model ($PrBa_{0.5}Sr_{0.5}Co_{1.5}Fe_{0.5}O_{6.0}$; $Pr_4Ba_2Sr_2Co_6Fe_2O_{24}$; P*4/mmm*) calculated at the GGA-PBE level. Optimized lattice constants are at a =b = 3.8728 Å, c = 7.5591 Å, Surface models of **b** CoFeO-terminated PBSCF (100), **c** CoO-terminated PBSCF (100), **d** PrBaSrCoFeO-terminated PBSCF (110), **e** PrBaSrCoO-terminated PBSCF (110), and **f** CoFe-terminated PBSCF (111). For simplicity, the surface models are referred to as CoFe- or Co-terminated (100), (110), and (111) surfaces, depending its inclusion of catalytically active elements of Co and Fe. **g** Representative adsorption configuration of O*, HO*, and HOO* on Co-terminated PBSCF (100).

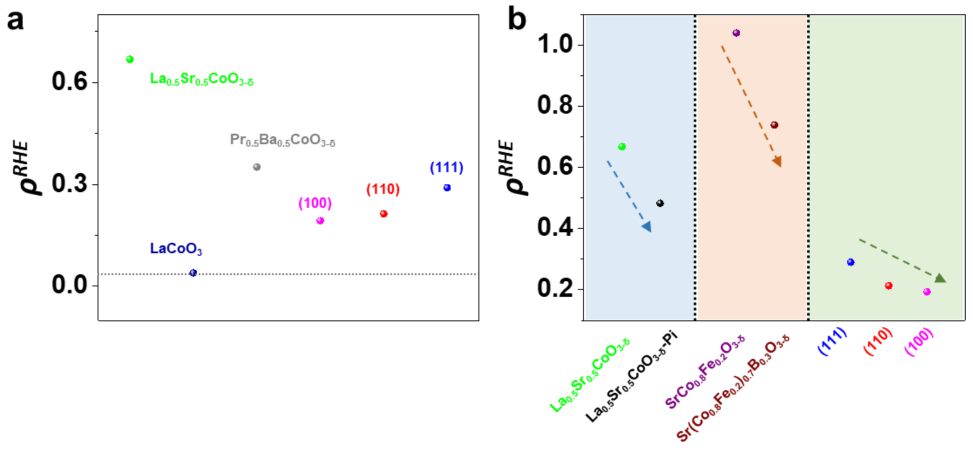

Supplementary Figure 12 ρ^{RHE} **reported for different perovskite materials.**[1-3]

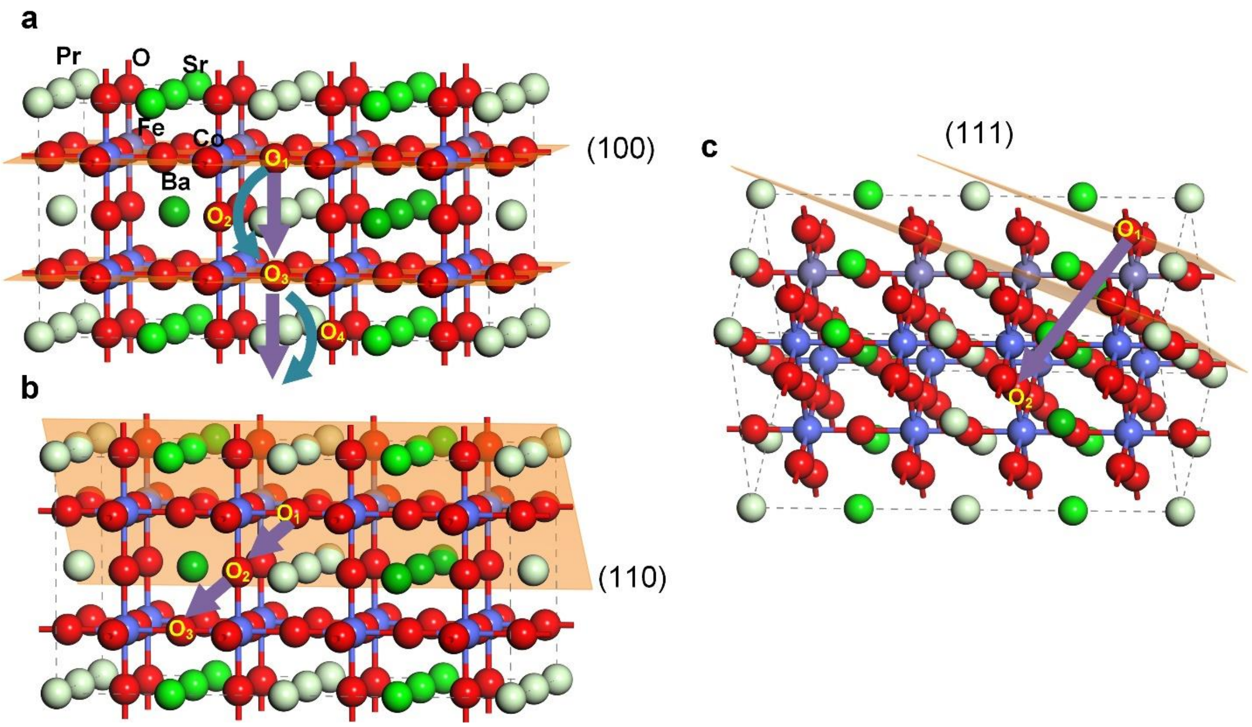

Supplementary Figure 13 Geometrical illustrations of bulk PBSCF along different orientations a [100] **b** [110] **c** [111] directions. Miller indices in parenthesis represent its crystal plane. The arrows in purple are the direction of bulk diffusion of proton or oxygen ions for migration energy calculations using the CI-NEB method.

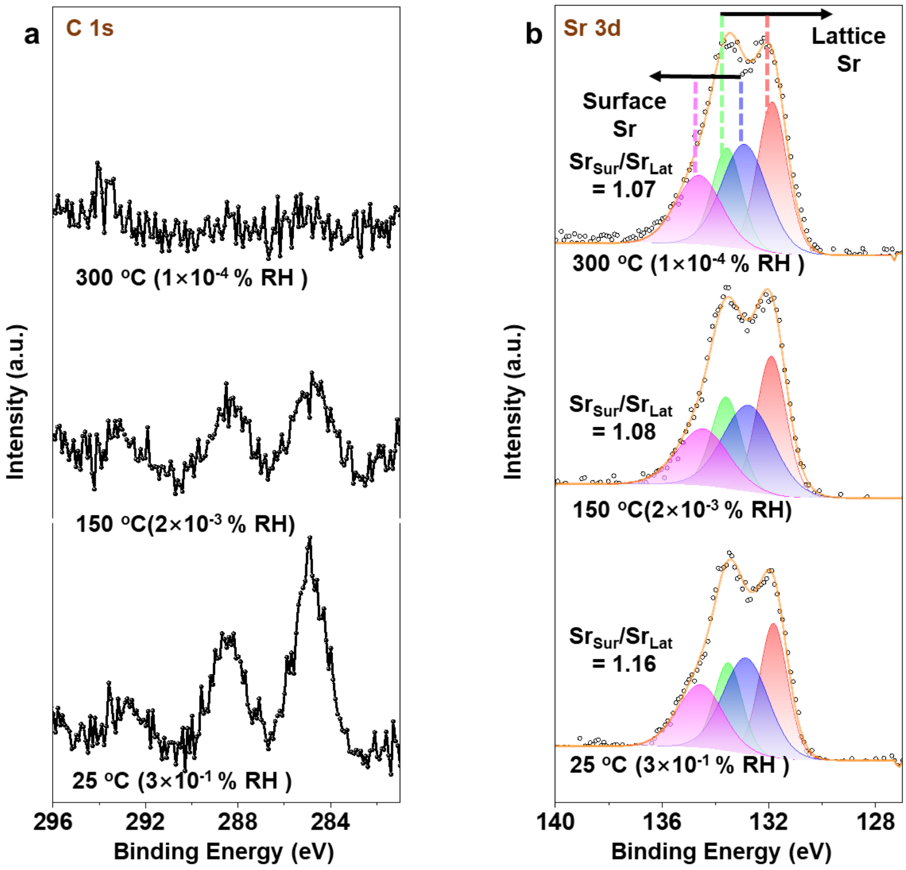

Supplementary Figure 14 XPS spectra for PBSCF thin films with different RH
a C 1s; **b** Sr 3d XPS spectra on the (100) film in 1 mbar H_2O at 300 °C (1×10^{-4} % RH), 150 °C (2×10^{-3} % RH), 25 °C (3×10^{-1} % RH).

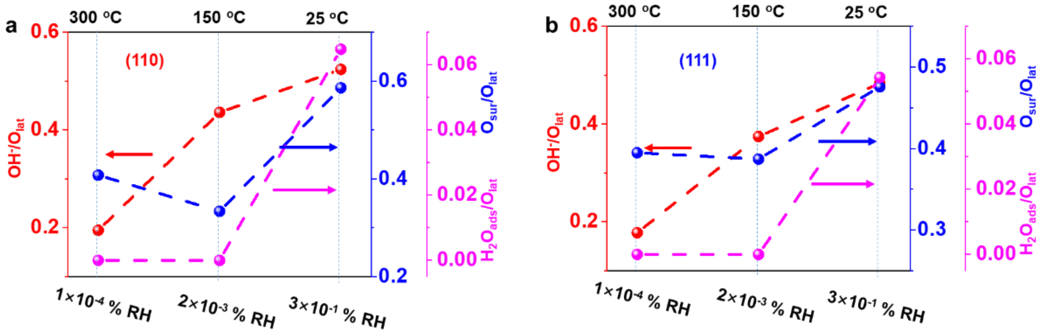

Supplementary Figure 15 Fitting results for HO^{-}/O_{lat}, O_{sur}/O_{lat} and H_2O_{ads}/O_{lat} ratio on PBSCF films a (110) and **b** (111) film in 1 mbar H_2O at 300 °C (1×10^{-4} % RH), 150 °C (2×10^{-3} % RH), 25 °C (3×10^{-1} % RH).

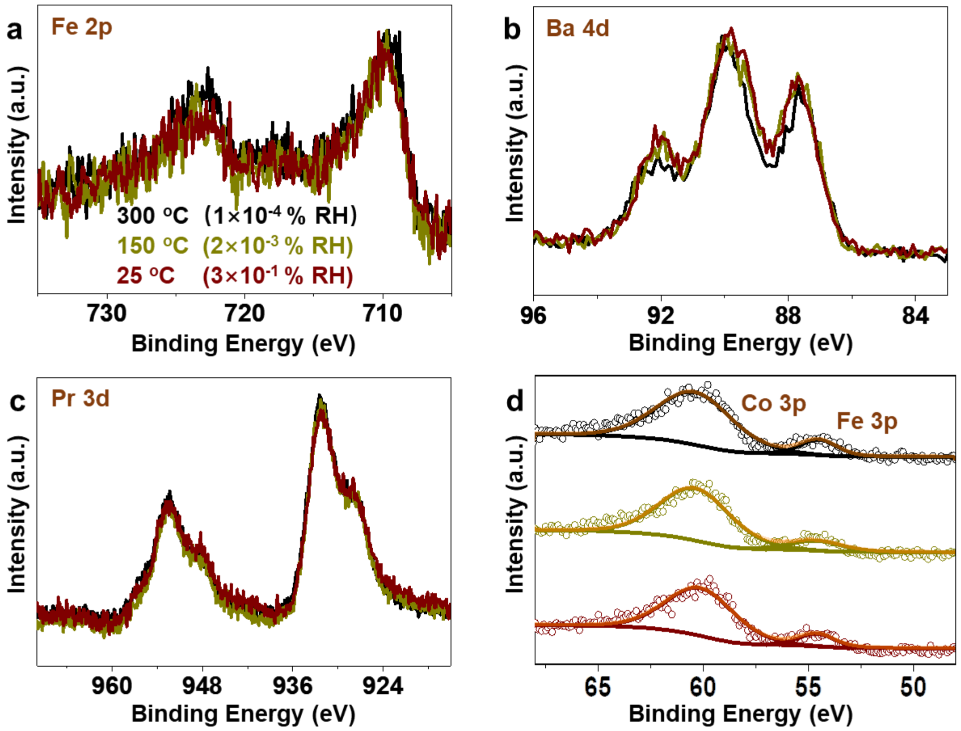

Supplementary Figure 16 XPS spectra on the (100) in different RH a Fe 2p; **b** Ba 4d; **c** Pr 3d; **d** Co 3p/Fe 3p XPS spectra on the (100) film in 1 mbar H_2O at 300 °C (1×10^{-4} % RH), 150 °C (2×10^{-3} % RH), 25 °C (3×10^{-1} % RH).

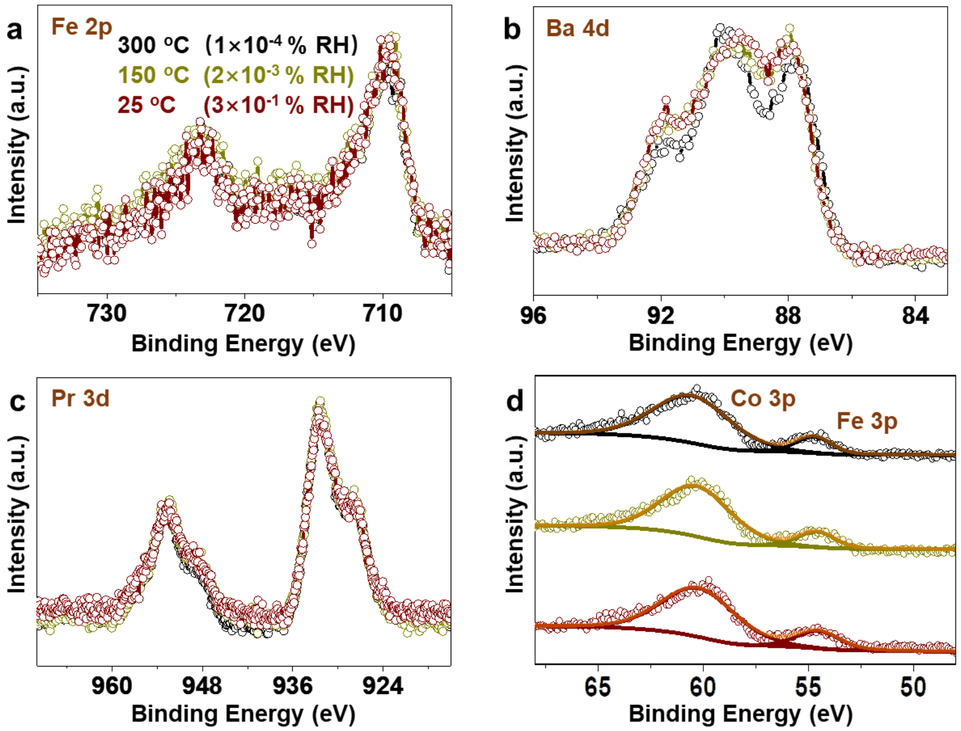

Supplementary Figure 17 XPS spectra on the (110) with different RH a Fe 2p; **b** Ba 4d; **c** Pr 3d; **d** Co 3p/Fe 3p XPS spectra on the (110) film in 1 mbar H_2O at 300 °C (1×10^{-4} % RH), 150 °C (2×10^{-3} % RH), 25 °C (3×10^{-1} % RH).

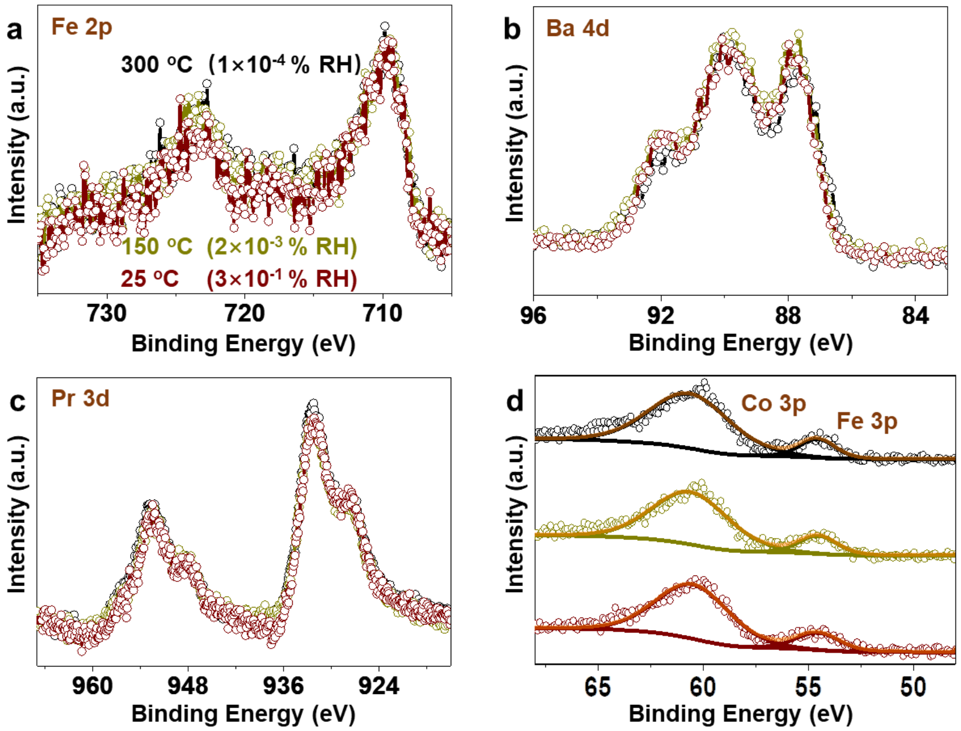

Supplementary Figure 18 XPS spectra on the (111) with different RH a Fe 2p; **b** Ba 4d; **c** Pr 3d; **d** Co 3p/Fe 3p XPS spectra on the (111) film in 1 mbar H_2O at 300 °C (1×10^{-4} % RH), 150 °C (2×10^{-3} % RH), 25 °C (3×10^{-1} % RH).

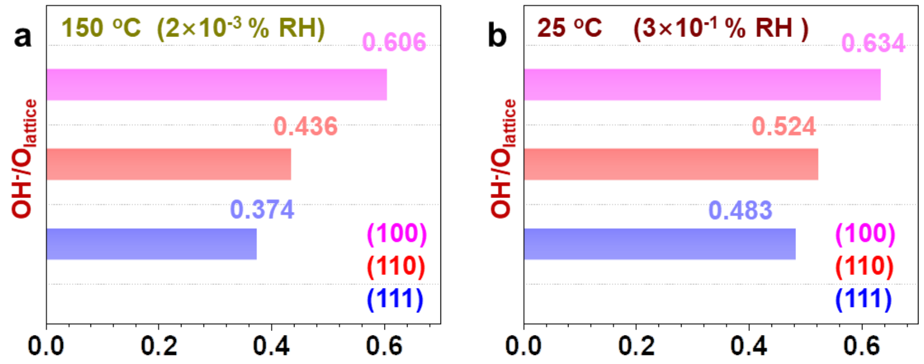

Supplementary Figure 19 HO^-/O_{lat} ratio for PBSCF thin films with different orientation a 150 °C (2×10^{-3} % RH), **b** 25 °C (3×10^{-1} % RH) in 1 mbar H_2O.

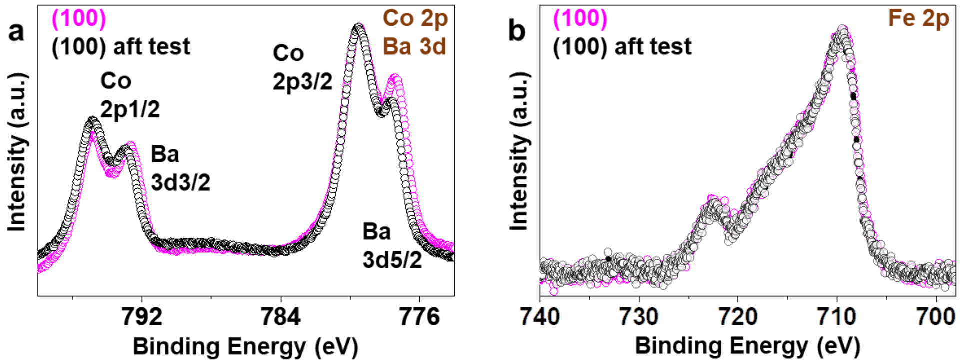

Supplementary Figure 20 XPS spectra of the (100) before and after test a Co 2p/Ba 3d and **b** Fe 2p

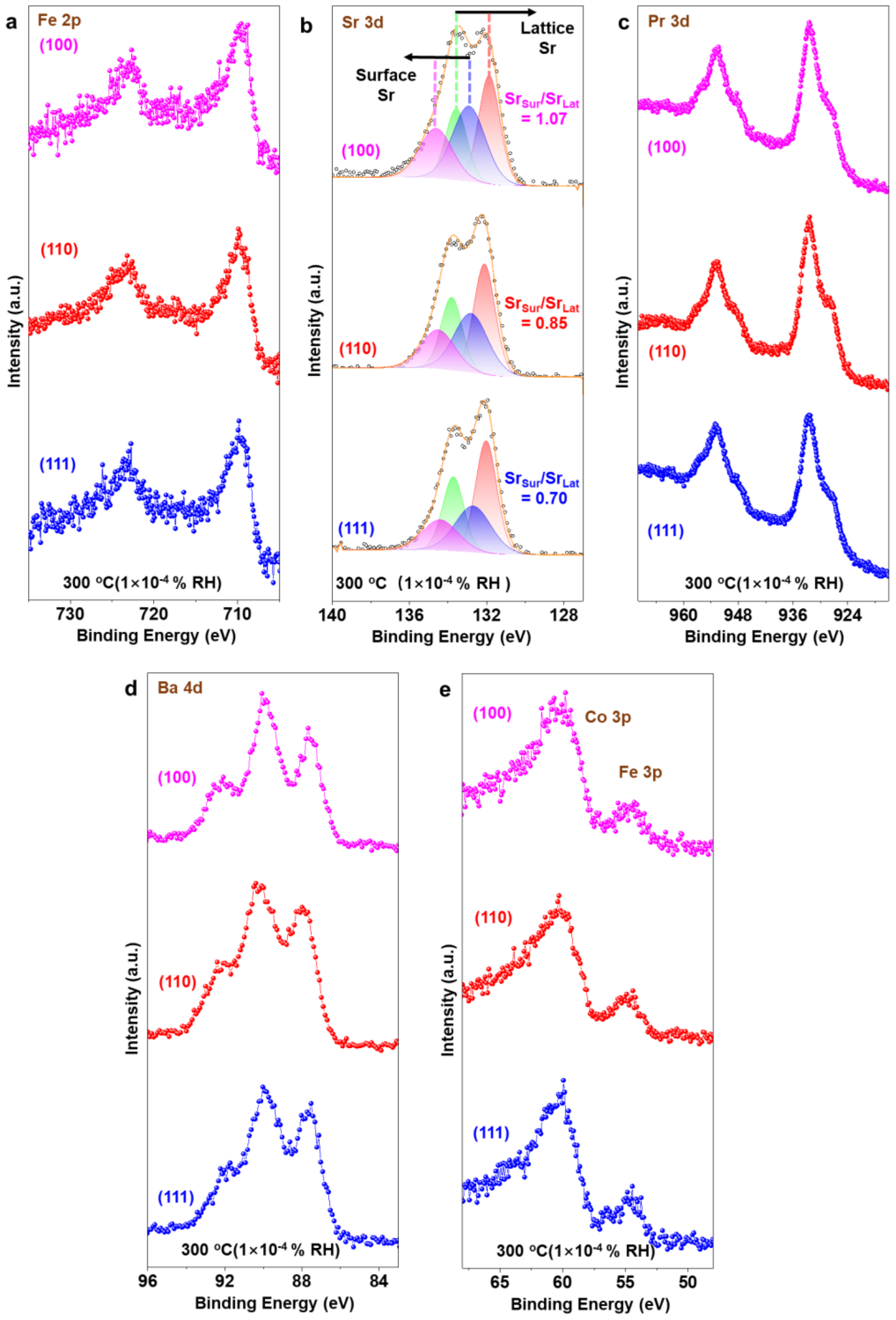

Supplementary Figure 21 XPS spectra of PBSCF thin films in different RH a Fe 2p, **b** Sr 3d, **c** Pr 3d, **d** Ba 4d, **e** Co 3p/Fe 3p spectra in 1 mbar H_2O at 300 °C (1×10^{-4} % RH).

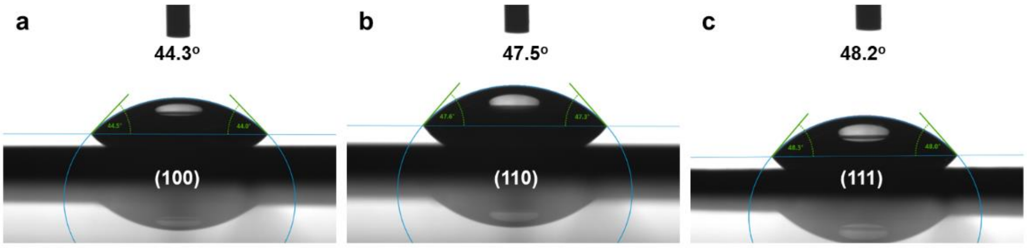

Supplementary Figure 22 Contact angle of electrolytes on PBSCF thin films
a (100), b (110) and c (111)

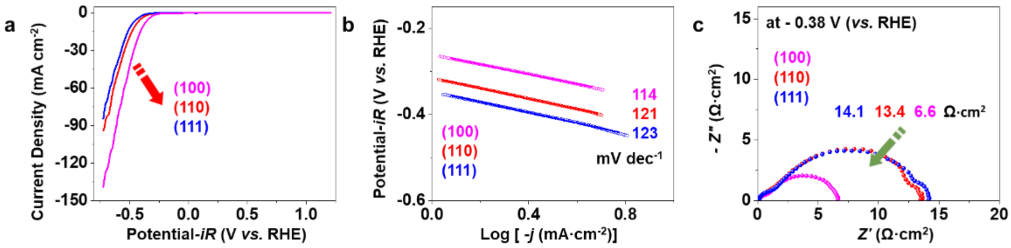

Supplementary Figure 23 HER activity for PBSCF thin films with different orientation a LSV curves; **b** Tafel plots; **c** Nyquist plots of HER on PBSCF thin film electrodes in 1M KOH at -0.38 V (vs. RHE).

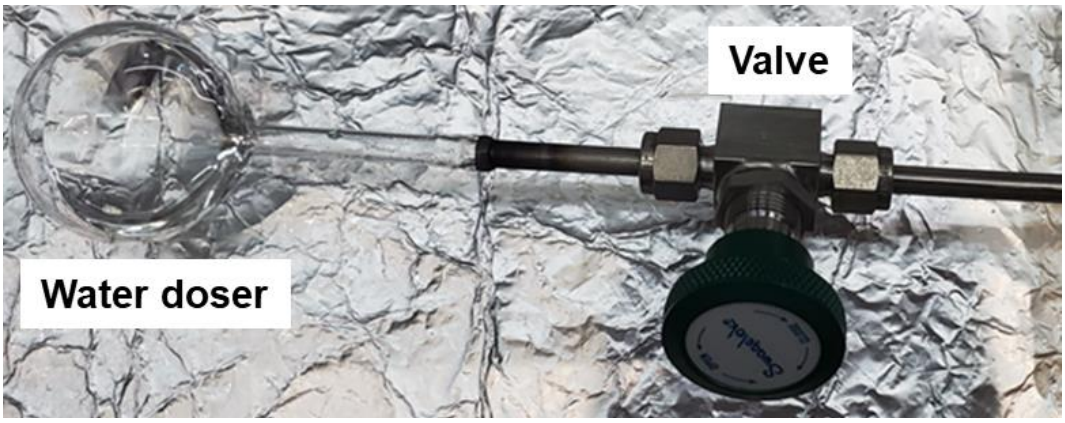

Supplementary Figure 24 Image of the water doser used in the APXPS measurements.

Supplementary Table 1 Compilation of calculated overpotentials and $\Delta G_{O*} - \Delta G_{HO*}$ on PBSCF (100), (110), and (111) surfaces.

Surface orientation	Termination	$\Delta G_{O*} - \Delta G_{HO*}$ (eV)	Theory (V)	Potential determining step
(100)	CoFe	1.46	0.23	G_2
(100)	Co	1.43	0.20	G_2
(100)	average	1.45	0.22	G_2
(110)	CoFe	1.18	0.69	G_4
(110)	Co	1.23	0.70	G_4
(110)	average	1.21	0.70	G_4
(111)	CoFe	1.13	0.83	G_3

Supplementary Table 2 Summary of the adsorption energies of O* and H* on PBSCF (100), (110), and (111) surfaces.

Surface orientation	Termination	$E_{ad,O*}$ (eV)	$E_{ad,H*}$ (eV)
(100)	CoFe	–3.48	–1.48
(100)	Co	–3.33	–1.64
(100)	average	–3.41	–1.56
(110)	CoFe	–4.84	–2.26
(110)	Co	–4.73	–2.36
(110)	average	–4.79	–2.31
(111)	CoFe	–5.09	–2.39

Supplementary Table 3 Migration barriers of E_m for the diffusion of protons and oxygen ions in PBSCF along different crystalographic directions.

Direction	E_m (eV) of H-diffusion[a]	E_m (eV) of O-diffusion [a]
[100]	0.31	1.15
[110]	0.55	1.50
[111]	1.00	5.39

[a] According to our examination of the trajectory from the CI-NEB, the diffusion may occur via a hopping via $O_1 \rightarrow O_2 \rightarrow O_3$ (see Supplementary Fig. 13).

Supplementary Note 1. Synthesis of PBSCF material and target.
$PrBa_{0.5}Sr_{0.5}Co_{1.5}Fe_{0.5}O_{5+\delta}$ (PBSCF) powder was synthesized using a nitrate combustion method. Correct molar ratio of metal nitrates $Pr(NO_3)_3 \cdot 6H_2O$, $Sr(NO_3)_2$, and $Co(NO_3)_2 \cdot 6H_2O$ (Alfa), $Ba(NO_3)_2$ and $Fe(NO_3)_3 \cdot 9H_2O$ (Sigma) and glycine (Sigma) were added into deionized water, and were stirred until all the chemicals were completely dissolved. The mixture was then heated until most of the water have evaporated. The obtained powder was ground and calcined in air at 400 °C for 1 hour to complete remove any nitrates, and then calcined in air at 1200 °C for 6 hours. To obtain the PBSCF target, PBSCF powder and an appropriate amount of Polyvinyl Butyral (PVB) were thoroughly mixed in an ethanol solution. The uniformly mixed powder was pressed into a target with a diameter of 25 mm. It was then calcined at 400 °C for 1 hour in air until the organic matter was completely volatilized, and then calcined at 1250 °C for 6 hours. The obtained the targets showed the characteristics peaks of PBSCF with double perovskite structure reported in literature (as shown in Supplementary Fig. 1b).

Supplementary Note 2. Electrochemical measurements.
The potential in this study is referenced to the RHE potential scale and subjected to *iR* correction as determined by the high frequency AC impedance (R_s). Electrochemical impedance spectroscopy (EIS) was carried out at 0.55 V, 0.66 V, 0.70 V, and 0.75 V versus Ag/AgCl for OER, at -1.2 V, -1.3 V, -1.4 V, and -1.5 V versus Ag/AgC. For the ionic diffusion measurement, 6 M KOH electrolyte was purged with N_2 for more than 1 hour to ensure that the electrolyte was saturated with N_2, and then a different bias voltage (50 and 100 mV) was applied to obtain a current-time response relationship.

Supplementary Note 3. Ambient pressure X-ray photoelectron spectroscopy measurements.
Deionized water (> 18.2 MΩ cm^{-1}) in a glass bottle (Supplementary Fig. 24, made of special glass) was frozen into ice with liquid N_2, followed by mechanical pumping < 5 × 10^{-7} to remove any impurity such CO_2 and O_2 gases dissolved in deionized water. The freezing and pumping procedure was repeated several times to purify water. Finally, water vapor was introduced into the analysis chamber with a controllable amount through a leak valve.

Supplementary Note 4. Density functional theory (DFT) calculations.

In order to systematically comprehend the mechanism of the OER on different surface orientations and to theoretically calculate overpotentials, we performed periodic density function theory (DFT) simulations. In this study, we applied the Vienna ab initio simulation package (VASP)[4] with the projector-augmented-wave (PAW) method.[5, 6] We also used the spin-polarization method with the Perdew-Burke-Ernzerhof (PBE)[7] exchange-correlation functional. To accurately support the experimental findings, we first prepared a bulk model for $PrBa_{0.5}Sr_{0.5}Co_{1.5}Fe_{0.5}O_6$ (PBSCF; $Pr_4Ba_2Sr_2Co_6Fe_2O_{24}$; *P4/mmm*) (Supplementary Fig. 11). A kinetic energy cutoff for a plane wave basis set of 415 eV was applied, while Monkhorst-Pack meshes[8] with the (3 × 3 × 3) and (3 × 3 × 1) were applied for bulk and surface calculations, respectively. Then eight-atomic-layer (100), (110), and (111) surface models for PBSCF ($Pr_8Ba_4Sr_4Co_{12}Fe_4O_{48}$) with a vacuum space of 15 Å were constructed to mimic the three thin-film orientations with (100), (110), and (111) (Supplementary Fig. 11). The dipole correction was applied to circumvent polarization in surface calculations. The adsorption of the intermediate species of O*, HO*, and HOO* was calculated on each surface model (Supplementary Fig. 11g). As schematically illustrated in Supplementary Fig. 11, PBSCF (100) and (110) surfaces have two catalytically active terminations (*i.e.*, CoFeO- and CoO-termination and PrBaSrCoFeO- and PrBaSrCoO-termination, respectively), while PBSCF (111) is CoFe-terminated. For simplicity, they are referred to as CoFe- or Co-termination. In this study, to minimize the potential error from the different surface size of PBSCF (100), (110), and (111) surfaces, its full coverage (1 monolayer; 1ML) was examined. The six-bottom layers were fixed to the bulk parameters, while the two-top layers and adsorbates were allowed to fully be relaxed. Similarly, the adsorption of H* was optimized. In this study, the adsorption energies of O and H were calculated by referencing to gas-phase O and H atom, respectively ($E_{ad,O*}$ and $E_{ad,H*}$, respectively, Supplementary Table 2). We precisely followed the standard approach to obtain theoretical overpotentials by calculating the Gibbs free energy of PBSCF (100), (110), and (111) surfaces using the electronic energies from DFT calculations.[9-11] Zero-point energies (ZPE), vibrational enthalpies (H_{vib}), and entropy contributions were included.[10, 11] In this study, we used the experimental H_2O formation energy according to $2H_2 + O_2 \rightarrow 2H_2O$, $\Delta G^0 = -4.92$ eV, while the calculated energies of H_2O and H_2 using DFT were applied. The Gibbs free energy change for the four steps were calculated using the following equations.

$$\Delta G_1 = \Delta G_{HO*} - eU + kT \cdot \ln a_{H+} \qquad \text{Equation 1}$$

$$\Delta G_2 = \Delta G_{O*} - \Delta G_{HO*} - eU + kT \cdot \ln a_{H+} \qquad \text{Equation 2}$$

$$\Delta G_3 = \Delta G_{HOO*} - \Delta G_{O*} - eU + kT \cdot \ln a_{H+} \qquad \text{Equation 3}$$

$$\Delta G_4 = 4.92\ (\text{eV}) - \Delta G_{HOO*} - eU + kT \cdot \ln a_{H+} \qquad \text{Equation 4}$$

where U is the measured potential versus NHE under standard conditions. After calculating the Gibbs free energies for the four-step reaction mechanism (*i.e.*, ΔG_1, ΔG_2,

ΔG_3, and ΔG_4), the largest G value is chosen according to G^{OER} = max [ΔG_1^0, ΔG_2^0, ΔG_3^0, ΔG_4^0] at U = 0, pH = 0, p = 1 atm, and T = 300 K. Then the theoretical overpotential is calculated by $\eta_{theo.} = (G^{OER}/e) - 1.23$ V. It is worth noting that discrepancies exist between the calculated and measured overpotentials because of the omitting of activation barriers in the theoretical analysis. Also, other factors (such as pH, surface active sites, and phases) have to be taken into consideration.[9]

In addition, in this study, to understand the migration phenomena and its associated migration barriers (or diffusion barriers, E_m), the climbing image nudged elastic band (CI-NEB)[12] was applied to locate the migration barriers. Our fully relaxed CI-NEB calculations sucessfully show that protons hop to the next stable point.

Supplementary References

1. Yang C., *et al.* Phosphate Ion Functionalization of Perovskite Surfaces for Enhanced Oxygen Evolution Reaction. *J. Phys. Chem. Lett.* **8**, 3466-3472 (2017).
2. Grimaud A., *et al.* Activating lattice oxygen redox reactions in metal oxides to catalyse oxygen evolution. *Nat. Chem.* **9**, 457-465 (2017).
3. She S. X., Zhu Y. L., Chen Y. B., Lu Q., Zhou W., Shao Z. P. Realizing Ultrafast Oxygen Evolution by Introducing Proton Acceptor into Perovskites. *Adv. Energy Mater.* **9**, 1900429 (2019).
4. Kresse G., Furthmuller J. Efficient iterative schemes for ab initio total-energy calculations using a plane-wave basis set. *Phys. Rev. B* **54**, 11169-11186 (1996).
5. Blochl P. E. Projector augmented-wave method. *Phys. Rev. B* **50**, 17953-17979 (1994).
6. Kresse G., Joubert D. From ultrasoft pseudopotentials to the projector augmented-wave method. *Phys. Rev. B* **59**, 1758-1775 (1999).
7. Perdew J. P., Burke K., Ernzerhof M. Generalized gradient approximation made simple. *Phys. Rev. Lett.* **77**, 3865-3868 (1996).
8. Monkhorst H. J., Pack J. D. Special points for Brillouin-zone integrations. *Phys. Rev. B* **13**, 5188-5192 (1976).
9. Man I. C., *et al.* Universality in Oxygen Evolution Electrocatalysis on Oxide Surfaces. *Chemcatchem* **3**, 1159-1165 (2011).
10. Ng J. W. D., *et al.* Gold-supported cerium-doped NiOx catalysts for water oxidation. *Nat. Energy* **1**, 16053 (2016).
11. Seitz L. C., *et al.* A highly active and stable $IrO_x/SrIrO_3$ catalyst for the oxygen evolution reaction. *Science* **353**, 1011-1014 (2016).
12. Henkelman G., Uberuaga B. P., Jonsson H. A climbing image nudged elastic band method for finding saddle points and minimum energy paths. *J. Chem. Phys.* **113**, 9901-9904 (2000).
